# Development and Testing of a Portable Virtual Reality-Based Mirror Visual Feedback System with Behavioral Measures Monitoring

**DOI:** 10.3390/ijerph19042276

**Published:** 2022-02-17

**Authors:** Beatriz Rey, Alejandro Oliver, Jose M. Monzo, Inmaculada Riquelme

**Affiliations:** 1 Departamento de Ingeniería Gráfica, Universitat Politècnica de València, 46022 Valencia, Spain; alolmol1@alumni.upv.es; 2 Instituto de Instrumentación para Imagen Molecular (I3M), Centro Mixto CSIC-Universitat Politècnica de València, 46022 Valencia, Spain; jmonfer@upvnet.upv.es; 3 Department of Nursing and Physiotherapy, University of the Balearic Islands, 07122 Palma, Spain; inma.riquelme@uib.es; 4 Institute of Health Sciences Research (IUNICS-IdISBa), University of the Balearic Islands, 07122 Palma, Spain

**Keywords:** mirror visual feedback, virtual reality, behavioral measures, response time, performance time, trajectories

## Abstract

Virtual Reality (VR) is a technology that has been used to provide the Mirror Visual Feedback (MVF) illusion to patients with promising results. In the present work, the goal is to design, develop and test a portable VR-based MVF system that monitors behavioral information about the performance of a simple motor task. The developed application runs in a stand-alone VR system and allows the researcher to select the real and virtual hands used to perform the motor task. The system was evaluated with a group of twenty healthy volunteers (12 men and 8 women) with ages between 18 and 66 years. Participants had to repetitively perform a motor task in four different experimental conditions: two mirror conditions (performing real movements with the dominant and with the non-dominant hand) and two non-mirror conditions. A significant effect of the experimental condition on embodiment score (*p* < 0.001), response time (*p* < 0.001), performance time (*p* < 0.001), trajectory length (*p* < 0.004) and trajectory maximum horizontal deviation (*p* < 0.001) was observed. Furthermore, a significant effect of the experimental moment (initial, middle and final parts of the training) on the performance time was observed (*p* < 0.001). These results show that the monitored parameters provide relevant information to evaluate the participant’s task performance in different experimental conditions.

## 1. Introduction

There are many different types of ownership illusions that can be applied to manipulate the corporal schema. One of them is the mirror visual feedback (MVF) illusion. In this technique, participants move one of their limbs while observing a mirror located in their midsagittal plane. The opposite limb is hidden by the mirror, so it cannot be visualized by the participant. Only the reflections of the limb that is moving are observed in the mirror. This configuration makes participants believe that the movements that are observed in the mirror are in fact from their opposite limb.

### 1.1. Mirror Visual Feedback

MVF therapy was first applied for patients with an amputated limb who suffered from phantom limb pain [1]. The MVF illusion makes them believe that their missing limb is still there and performing the movements. Posterior studies have shown that the technique appears to be effective in controlling phantom limb pain, reducing the intensity and length of pain episodes [2]. Several controlled studies found these kinds of positive effects when the procedure was applied for several weeks [3,4,5]. The technique is simple and inexpensive, making it suitable for application at the patient’s home. In fact, the use of home-based self-delivered MVF has also shown reductions in phantom limb pain [6]. However, there are several issues related to the application of the technique that remain open. It has been observed that individual differences have an important influence on the MFV procedure effectiveness [7]. Consequently, further studies are needed to evaluate the role of individual factors on the treatment outcomes, to standardize protocols and to personalize them according to the patients’ characteristics. Besides, there is a need to improve methodological aspects in these studies, increasing the sample sizes, improving the experimental designs and evaluating medium and long-term effects [2].

The MVF illusion has also been applied to other pain patients, such as those with complex regional pain syndrome [8,9,10] and seems to be also appropriate for chronic pain patients [11]. On the other hand, it has also been evaluated for the rehabilitation of stroke patients, to help them to recover motor function in the affected limbs [12,13,14,15,16,17]. Further studies are also needed in this case to analyze the long-term effects and impact on daily activities of the procedure.

### 1.2. Virtual Reality

Virtual Reality (VR) is a technology that can be used to immerse participants in a virtual world where they are represented by virtual avatars. It has been proposed to provide the visual feedback needed in the MVF illusion [18,19], instead of using a mirror as in the traditional procedure.

In the real world, people experience embodiment in their own real body [20]. They are inside their body and control it. However, in the virtual environment, the person’s body is substituted by a virtual one, generating similar sensations towards the virtual body. Three subcomponents are identified as fundamental for this sense of embodiment: self-location, body ownership and agency [21]. The sense of self-location implies that the volume where the person feels to be located is the volume occupied by the virtual body. The sense of body ownership means that people have the perception that they own the virtual body or a part of the virtual body, and that this virtual body is the origin of their sensations [22]. Finally, the sense of agency implies that people recognize themselves as the actors that generate the movements of their virtual body [23].

### 1.3. Virtual Reality Based Mirror Visual Feedback

As VR is a technology that can be used to generate embodiment in a virtual body, it is suitable to provide the visual feedback required for manipulations of the corporal schema. In the MVF procedure, the movements of one limb are monitored by tracking devices, and this information is used to change the position and rotation of the opposite limb in a mirror way.

As happened with the traditional MVF, VR-based MVF therapy has been applied for stroke patients [24], phantom limb pain [19,25] and complex regional pain syndrome [26]. The technique has also been applied in combination with other VR techniques such as exposure therapy for patients with functional neurological disorders [27].

Previous studies with VR-based MVF have found positive effects after applying the rehabilitation procedure, including a reduction in the level of pain and improvement in motor skills [18]. The majority of published studies are case studies or case series [19].

Most of the VR-based MVF studies are focused on upper limb rehabilitation. In some cases [27], the virtual representation shows both hands: the healthy hand and the mirror hand that follows the movements of the healthy one in a mirrored way. In other cases [28,29], only the mirror hand is shown moving in the virtual environment. A reduced number of studies have applied the technique to lower limb rehabilitation [30,31]. Although Miclaus et al. [32] applied VR exergaming training combined with traditional MVF in a recent study for lower extremity rehabilitation, the MVF training was not included in VR.

It is interesting to remark that some studies have evaluated the MVF procedure in healthy participants with different purposes. Mazzola et al. [28] evaluated if the mirroring training was associated to a higher similarity in activation between the dominant hand (which was physically moved during the procedure) and the non-dominant hand (which remained static during the procedure) in comparison with non-mirror training. They found significant differences in muscle activity between conditions for the dominant arm. Ossmy and Mukamel [33] evaluated the transfer of motor skills from one hand to the opposite one and found enhanced within-session performance gains in the hand that was not really moved during the procedure.

In general, behavioral measures about the performance during the training procedure are not collected during VR-based MVF studies. We can point out that Mazzola et al. [28] recorded time measurements associated to task completion, as well as position and rotation data of relevant environment elements. However, of all the collected behavioral data, only the time measurements were included in the final analyses. Swee at al. [34] also present in their experimental results the time upon completion of a pick and place task that was repeated thrice by the participants during the testing. Ossmy and Mukamel [33] describe a customized software used to read glove recorded data files, which allows researchers to visualize the recorded movements after finishing the training, to evaluate success and calculate performance gains.

### 1.4. Goals

The main goal of the present work is to design, develop and test in healthy participants a versatile and portable VR-based MVF system, suitable for home-based training that monitors behavioral information (temporal responses and trajectories) about the participant’s performance. Two factors are fundamental to allow participants to perform the VR-based MVF motor training remotely. On one hand, the VR hardware configuration should be simple and portable. On the other hand, it is especially important that the system monitors behavioral parameters about the participant’s performance, so this information can be accessed remotely by the trainer to evaluate the participant’s evolution during the training.

The testing will be conducted with healthy volunteers to analyze if the monitored parameters provide relevant information to evaluate the participant’s performance, which will support the validation of the system with patients in future studies. The two secondary goals of the testing are: (1) to analyze if the difficulty level of different configurations (mirror, non-mirror, using dominant hand, using non-dominant hand) of the developed system has an influence on the temporal responses and trajectories and (2) to evaluate if the repetitive performance of the training task during the experience has an influence on the temporal responses and trajectories. The initial hypotheses are that both the experimental condition and the repetitive training would have an influence on the behavioral parameters.

## 2. Materials and Methods

In the following paragraphs, the technical details of the developed system and the procedure used for its validation are described.

### 2.1. Technical Aspects of the System

The developed system allows the participant to perform a simple motor task in a VR environment. The system is versatile and allows different options for the task performance (such as mirror/non-mirror performance, one/two hands visualization and configurable number of repetitions) that can be modified depending on the experimental goals. In the following subsections, details about the technical features of the developed system are given.

#### 2.1.1. Hardware

The HMD that has been selected to present the visual information to the participant is the Meta Quest. This device works as a stand-alone VR system, which does not require any additional hardware to work. The position and orientation of the device is tracked with 6 degrees of freedom by means of an inside out optical system, so no external cameras are needed.

**Figure 1:**
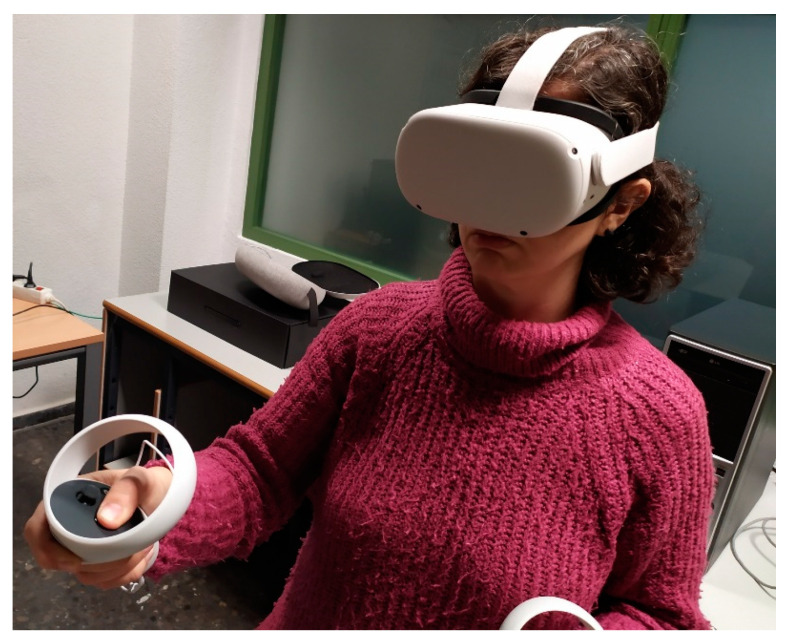
Person using the hardware system: Meta Quest head mounted display and controllers.

A person using the complete system (head mounted display and controllers) is shown in Figure 1.

The HMD comes with two controllers. Each controller has several action buttons, a grip button, a trigger button, as well as an analog thumb stick. Besides, they have a wrist strap so controllers can be held to the wrists during use to avoid dropping them involuntarily. The controllers’ position and orientation are also tracked with 6 degrees of freedom with an inside out optical system. The controllers also include sensors able to detect if the fingers are resting on specific buttons allowing the detection of gestures based on the fingers’ location.

#### 2.1.2. Software

The Unity real-time 3D engine has been used to develop the VR application. Oculus Integration tools are used to program all the aspects related to the communication and control of VR devices (head mounted display and controllers).

#### 2.1.3. Virtual Environment

**Figure 2:**
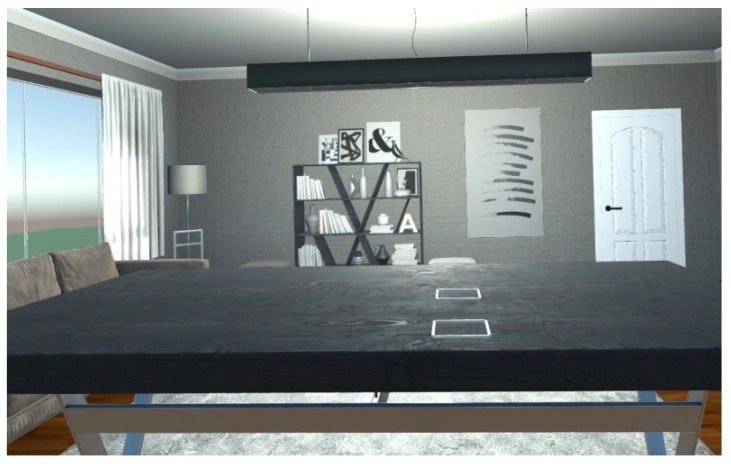
Virtual environment used for the application.

The virtual environment is a house room with a window and a door. There is furniture distributed through the room, including two tables, a sofa and some shelves with books. The user will visualize the environment from a first person (egocentric) point of view, in front of the bigger table. The environment can be visualized in Figure 2.

#### 2.1.4. Interaction with the Virtual Environment

The interaction with the virtual environment is limited to specific actions. By default, the system updates the user’s point of view according to the tracked head movements, and the hands position and orientation according to the tracked hands’ movements. Besides, depending on the fingers’ position, some gestures are detected and shown in the virtual hands of the 3D environment. For example, the hand will be opened if the grip button, the trigger button and the thumb stick are not being touched. The index finger will be pointing forward if just the trigger button is not being touched. The hand will be closed, like a fist, if all the indicated buttons are being touched.

Apart from these generic responses that are provided by default by the Oculus Integration tools, the Unity application has been programmed for additional interactions.

Firstly, it is possible to grab specific objects of the environment. If the virtual hand is located close to a selectable object in the environment and the user presses the trigger button, the grip button, or both simultaneously, the object will be grabbed and will appear held by the hand. It can be thrown away at any moment just by opening the hand.

Secondly, the mirror behavior has also been programmed. When the application is working with MVF, users will use just one controller with one of their hands, and the opposite virtual hand will follow the real hand movements in a mirror way with respect to the mid-sagittal plane. The coordinates origin is located in the mid-sagittal plane and the x axis is defined as perpendicular to this plane. In order to obtain the mirror hand position, the sign of the real hand’s x value is changed, and the y and z values take the same value as in the real hand. Regarding the rotation, the mirror hand local rotation coordinates take the same absolute values as the real hand local rotation, but the sign of the rotations about the y and z axes is changed. Besides, any gesture that is performed by the real hand will be replicated by the virtual hand in a mirrored way.

#### 2.1.5. Motor Task

**Figure 3:**
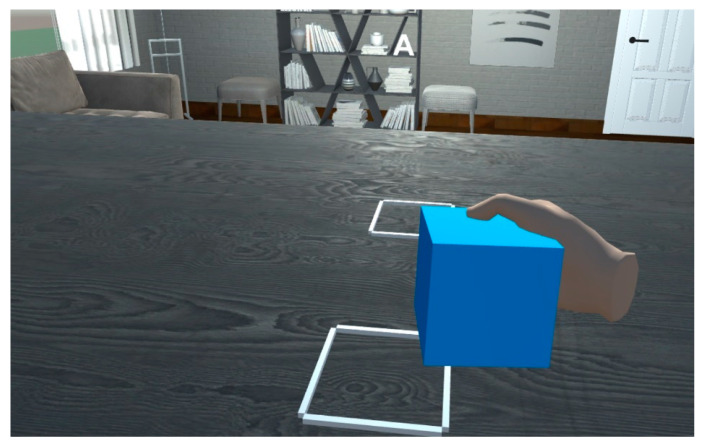
Exercise performance. Moving the cube from the initial to the final location.

The application allows the performance of a simple motor task in a repetitive way. A cube will appear on the table in specific moments of the application execution. When the cube appears, the subject must grab the cube and move it from its initial location to a final location that is marked in the table, as can be observed in Figure 3.

#### 2.1.6. Configuration Parameters

The configuration parameters for the training procedure are included in an xml input file that the application reads. An xml file is a text file that uses the Extensible Markup Language (XML) for storing arbitrary data in an organized way. This language uses tags to represent the data structure and stores the data within the tags. For the purposes of the present work, several tags are used to contain the different configuration parameters, which include:

- The real hand: left or right. This parameter indicates the real hand that is going to be used to perform the exercise.
- The virtual hand: left, right, or both. This parameter indicates the hand or hands that are going to be visualized in the virtual world. If the virtual hand coincides with the real one, no mirrored effect will be observed. If the virtual hand is the opposite to the real one, the mirror effect will be applied to the real hand movements to obtain the virtual hand movements. If both hands are visualized, the hand that coincides with the real one will follow the real movements and the opposite one will follow the real movements in a mirror way.
- Initial waiting period. An initial waiting period in seconds is indicated. During this initial period, no cube appears on the table, so the user has no task to perform
- Timing information for the task repetitions. For each repetition, the maximum time available to perform the task is indicated. In the initial moment of each repetition, a cube will appear on the table, and the participant will have to move it to its final position. If the user leaves the cube when it is in the final location, the task will be successful, and the cube will disappear. However, if the user leaves the cube before arriving to the target location, the cube will return to the original point, so the exercise can be tried again if there is still time available. If the maximum time to perform the task has elapsed, and the task has not been successfully finished, the cube will disappear, and the next repetition will start. A new cube will appear in the initial location.

These parameters can be modified in the input xml file depending on the goals. The session will finish when all the repetitions defined in the input file have been performed.

#### 2.1.7. Behavioral Data

The VR application will monitor information about the participant’s behavior and performance during the different repetitions of the training task. This information will be saved in an output xml file, also with different tags used to structure the behavioral information about the performance. These parameters will be saved for each repetition:

- Iteration number.
- Successful iteration (Yes/No). This parameter lets us know if the current repetition task has been successfully performed.
- Reaction time(s). Time until the cube is first grabbed since its appearance.
- Performance time(s). Time that the participant needs to move the cube from its initial position to its final one. If the repetition is successful, it will be the elapsed time since the cube is grabbed for the first time in the iteration until it is left in its correct final location. If the repetition is not successful, the reaction time plus the performance time will be equal to the maximum time available to perform the task.
- Trajectory. List of coordinates (X, Y, Z) followed by the cube during its trajectory while it is held by the hand. From this data, the total length and the maximum deviations in the horizontal and vertical axes are calculated.

### 2.2. System Testing

The developed system was tested to analyze the effects of the mirror configuration (mirror, non-mirror, using dominant hand, using non-dominant hand) and the repetitive performance of the training task on the temporal responses and trajectories monitored during the performance. The participants’ demographic data, the specific configurations of the developed system that are used in the validation protocol and the statistical analyses procedure are described in the following subsections.

#### 2.2.1. Participants

Twenty healthy volunteers participated in the study (12 men and 8 women), with ages between 18 and 66 years (mean age 42 years, standard deviation 14.3 years). They were recruited by means of announcements on university notice boards. Inclusion criteria were to be healthy adults with ages between 18 and 70 years. All of the participants signed a written informed consent to participate in the study.

#### 2.2.2. Protocol

The developed system is versatile and allows different configurations for the training task. In the testing protocol, several configurations with different difficulty levels will be evaluated to analyze if they have an influence on the temporal responses and trajectories needed to perform the task. Each participant had to perform the VR training task in a repetitive way in four configurations with different combinations of virtual and real hands. These were the four experimental conditions included in the protocol:

- Dominant Real, Dominant Virtual (DR_DV). This configuration corresponds to motor training without MVF with the dominant hand. The participants must perform the movements in the real world with their dominant hand. In the virtual environment, the movements are also performed with the dominant hand. The non-dominant virtual hand is not shown in the virtual environment.
- Non-Dominant Real, Non-Dominant Virtual (NDR_NDV). This configuration is analogous to the previous one (motor training without MVF), but the non-dominant hand is used to perform the task.
- Dominant Real, Non-Dominant Virtual (DR_NDV). This configuration corresponds to a VR-based MVF training. The participants must perform the movements in the real world with their dominant hand. These movements will be visualized in the virtual environment in a mirror way in the non-dominant virtual hand.
- Non-Dominant Real, Dominant Virtual (NDR_DV). This configuration is analogous to the previous one (VR-based MVF training) but, in this case, the participant must perform the movements in the real world with the non-dominant hand. These movements will be visualized in the virtual world in the dominant hand in a mirror way.

The experimental conditions were presented to each participant in a random order.

The training procedure with the VR application for each configuration required the performance of the simple motor task previously described (moving a cube from an initial to a final position) in a repetitive way. The number of repetitions in each training procedure was selected so it was sufficiently high to allow learning and not too large to limit the global training time. Specifically, 15 repetitions of the motor training task were used. The participants had a maximum time to perform the task for each repetition. This time was selected to be enough to perform the task, but without an excessive temporal separation between repetitions, in the range between 10 s and 15 s. The exact time allowed for each repetition was randomly obtained. The exact length of the training procedure (15 repetitions) for each experimental condition depended on this random time assignation. It was in the range between 2 min 57 s and 3 min 12 s.

During the training, behavioral data about each repetition were monitored. Temporal parameters for the repetition included the response time and the performance time for the training task. Trajectory parameters were calculated from the hand trajectory data for that repetition: trajectory length, maximum horizontal deviation and maximum vertical deviation.

Three different temporal windows were considered in the study for analyzing the temporal evolution of the parameters: initial, middle and final parts of the training. The initial window parameters were calculated as an average of the parameters corresponding to the first five initial repetitions of the task. Analogously, the five central repetitions parameters were averaged to obtain the middle window parameters, and the five final repetitions parameters were averaged to obtain the final window parameters.

The study was conducted in a university laboratory, in the presence of one of the researchers. Before starting the procedure, the participant had to read a study information sheet. Then, demographic data (age, sex) about the participant were collected. Laterality was evaluated with the Edinburgh handedness inventory [35,36]. The participant also answered the Motion Sickness Susceptibility Questionnaire Short-form (MMSQ-Short) to evaluate susceptibility to motion sickness caused by different stimuli [37].

Once the participant had answered the questionnaires, the researcher explained how to interact with the VR environment in the different experimental conditions to the participant. The participant adjusted the head mounted display on the head (with the help of the researcher, if needed) and held the appropriate controller with the real hand corresponding to the first experimental condition. The experimental condition started when the participant pressed a button on the controller. Then, the cube appeared in a specific location on the table and the participant had to move it to its final location. This procedure was repeated 15 times as previously described. Once the 15 repetitions had been performed, the experimental condition finished and the participant gave the experimenter the controller and the head mounted display. Then, the participant answered the preliminary Embodiment Short Questionnaire (pESQ) to evaluate the sense of embodiment during that experimental condition, with the selected combination of real and virtual hands, focusing on three factors: self-location, agency and body ownership [38]. Besides, a short questionnaire based on previous studies [39] was included to evaluate if participants had had any positive (fun, relaxing, challenging, distracting or others) or adverse (dizziness, fatigue, anxiety or others) reactions during the experimental condition. A positive reaction was counted for participants that had at least one of the possible positive reactions, and a negative reaction if at least one of the possible adverse reactions have been marked.

Once these questionnaires had been answered, the participant continued with the next experimental condition and its associated questionnaires (pESQ and reaction questionnaires). The procedure was performed four times corresponding to the four experimental conditions included in the study.

When the trainings corresponding to the four experimental conditions had finished and their associated questionnaires had been answered, global presence in the virtual environment was evaluated by means of the SUS questionnaire [40], which is a questionnaire with six 7-point Likert-scale questions. Two parameters were calculated to evaluate the level of presence from the responses to this questionnaire: SUS Mean and SUS Count. SUS Mean was calculated as the mean of the responses to the six questions included in the questionnaire, and SUS Count was obtained as the number of ‘6’ or ‘7’ scores observed in the six questions [41]. An open answer question was included at the end of the presence questionnaire to allow the participants to express any comments they had about the system and the presence they had felt during the experience. The usability of the system was also evaluated using the System Usability Scale [42,43], which is a scale that evaluates the subjective opinion from a user regarding the usability of a particular device or tool.

The duration of the complete experimental session was approximately 30 min, including the exposure to the virtual environments and the evaluations with questionnaires.

#### 2.2.3. Statistical Analysis

All the statistical analyses were conducted using SPSS 16.0 (SPSS Inc., Chicago, IL, USA) with a 0.05 significance level.

The hypothesis of normality was not supported for questionnaire data (*p* < 0.05 in the Kolgomorov–Smirnov test), so in order to analyze the influence of the experimental condition (DR_DV, NDR_NDV, DR_NDV, NDR_DV) on the pESQ embodiment values, a nonparametric test, the Friedman Test, was applied. Post-hoc analyses with Wilcoxon Signed-Rank test were conducted with Bonferroni correction, resulting in a significance level set at *p* < 0.0083. In the case of binary variables, such as the positive reaction and the adverse reaction variables, which only have two possible values (yes/no), Cochran’s Q test was applied to analyze the effect of the experimental condition.

In the case of temporal parameters (response and performance times) and trajectory data (length and maximum horizontal and vertical deviations), two-way ANOVAs with repeated measures were applied to analyze the effects on these variables of the within-subject factors: experimental condition (DR_DV, NDR_NDV, DR_NDV, NDR_DV) and temporal moment (initial, middle and final periods). The assumption of sphericity of variables was checked using the Mauchly’s test, and if it was violated, Greenhouse–Geisser corrections were applied. Bonferroni correction was applied for pairwise comparisons.

## 3. Results

The virtual environment has been developed and its functionality tested. The system allows researchers to collect the previously indicated behavioral data about the performance (response and performance times, trajectories) of different repetitive tasks. Furthermore, the developed application can be run in a stand-alone head mounted display, so it is suitable to be used in the context of a home-based training, without needing any additional devices.

In the following subsections, the results of the validation of the system are summarized.

### 3.1. Handedness

Eighteen of the participants were right-handed, with Edinburgh handedness inventory values between 0 and 25 (Laterality Quotient between 40 and 100). The other two were left-handed. Their Edinburgh handedness inventory values were 40 and 42 (Laterality Quotients of −60 and −100, respectively).

### 3.2. MMSQ-Short

Eighteen of the participants reported low values in the MMSQ-Short (values between 0 and 13, percentiles lower than 55). Two participants reported higher values in the MMSQ-Short (values of 23 and 24, percentile values of 83.3 and 85.2), although this result was not associated to dizziness in the experience.

### 3.3. Embodiment (pESQ)

Median values of the embodiment values in the different experimental conditions are indicated in Table 1.

Results from the Friedman test show a significant effect of the experimental condition on the embodiment (pESQ global score), χ^2^ (3) = 23.998, *p* < 0.001. There were no significant differences between the two non-mirror conditions, DR_DV and NDR_NDV (Z = −1.604, *p* = 0.109) nor between the two mirror conditions, DR_NDV and NDR_DV (Z = −0.256, *p* = 0.798). On the other side, there were statistically significant differences between all combinations of mirror and non-mirror conditions: DR_NDV and DR_DV (Z = −3.186, *p* = 0.001), NDR_DV and DR_DV (Z = −2.942, *p* = 0.003), NDR_NDV and DR_NDV (Z = −2.821, *p* = 0.005), and NDR_NDV and NDR_DV (Z = −2.856, *p* = 0.004).

Results from the Friedman test for the self-location subcomponent of embodiment also show a significant effect of the experimental condition, χ^2^ (3) = 27.411, *p* < 0.001. Post-hoc tests showed significant differences between all combinations of mirror and non-mirror conditions (for all of them, Z ≤ −3.135, *p* ≤ 0.002). Besides, no significant differences were found between the two non-mirror conditions (NDR_NDV and DR_DV) nor between the two mirror conditions (DR_NDV and NDR_DV). For both cases, Z ≥ −0.903, *p* ≥ 0.367.

Results from the Friedman test for the agency factor also show a significant effect of the experimental condition, χ^2^ (3) = 12.515, *p* = 0.006. However, post-hoc tests did not show statistically significant differences between pairs of experimental conditions.

Results from the Friedman test for the body ownership factor show no significant effect of the experimental condition, χ^2^ (3) = 3.6, *p* = 0.308.

### 3.4. Positive and Adverse Reactions

The percentages of each type of positive reaction (fun, relaxing, challenging, distracting, others) and adverse reaction (dizziness, fatigue, anxiety, others) are shown in Table 2. Cochran’s Q test showed a statistically significant difference between the four experimental conditions in the proportion of participants that had an adverse reaction, χ^2^ (3) = 10.2, *p* = 0.017. No differences between conditions were found in the proportion of participants that had a positive reaction (χ^2^ (3) = 3.55, *p* = 0.318).

### 3.5. Response Times

Results from the two-way repeated measures ANOVA applied to response times show a significant effect for the experimental condition (F (3,42) = 15.215; *p* < 0.001). No significant effect was found for the other two factors (moment and interaction term).

**Figure 4:**
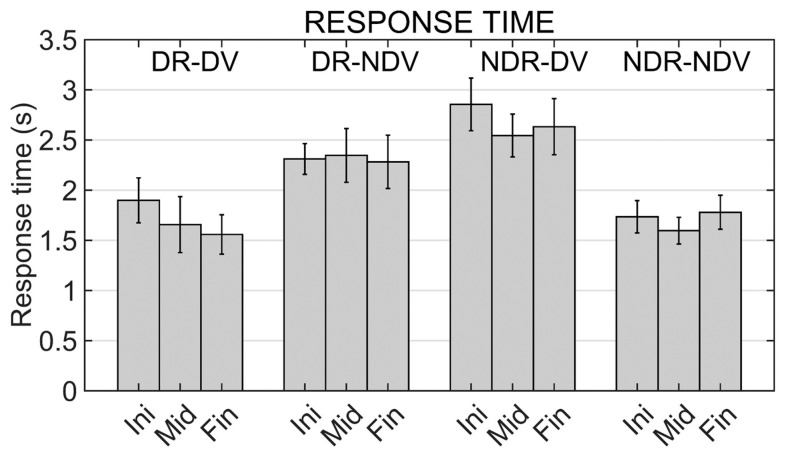
Response times in the different experimental conditions (DR_DV, DR_NDV, NDR_DV and NDR_NDV) and in the different moments of the training (Initial, Middle and Final moments). Mean values and standard errors of the mean are shown.

Pairwise comparisons using the Bonferroni correction showed significant differences in all comparisons between a mirror condition and a non-mirror condition: DR_DV and DR_DNV (*p* = 0.012), DR_DV and NDV_DV (*p* = 0.001), NDR_NDV and DR_NDV (*p* = 0.045), and NDR_NDV and NDR_DV (*p* < 0.001). Mean response times in the different experimental conditions and moments are shown in Figure 4.

### 3.6. Performance Times

Results from the two-way repeated measures ANOVA applied to response times show a significant effect for the experimental condition factor (F (2.113,29.586) = 19.693; *p* < 0.001) and for the moment factor (F (3,42) = 10.694, *p* < 0.001). A close to significant effect was also found for the interaction term (F (3.142,43.993) = 2.721, *p* = 0.053).

Pairwise comparisons between experimental conditions showed significant differences between DR_DV and DR_DNV (*p* < 0.001), DR_DV and NDR_DV (*p* = 0.002), NDR_NDV and DR_NDV (*p* = 0.001), and NDR_NDV and NDR_DV (*p* = 0.001), which include all the comparisons between a mirror condition and a non-mirror condition.

If the analysis between experimental conditions is performed separately in the three temporal moments of the experience, significant differences between mirror and non-mirror conditions are also observed in the initial moment (*p* < 0.009) and in the middle moment (*p* < 0.003). In the final moment, significant differences are only observed between the non-mirror conditions and the NDR_DV condition (*p* < 0.007).

Pairwise comparisons between moments showed significant differences between the initial part of the experiment and the middle part (*p* = 0.015) and between the initial part of the experiment and the final part (*p* = 0.006).

**Figure 5:**
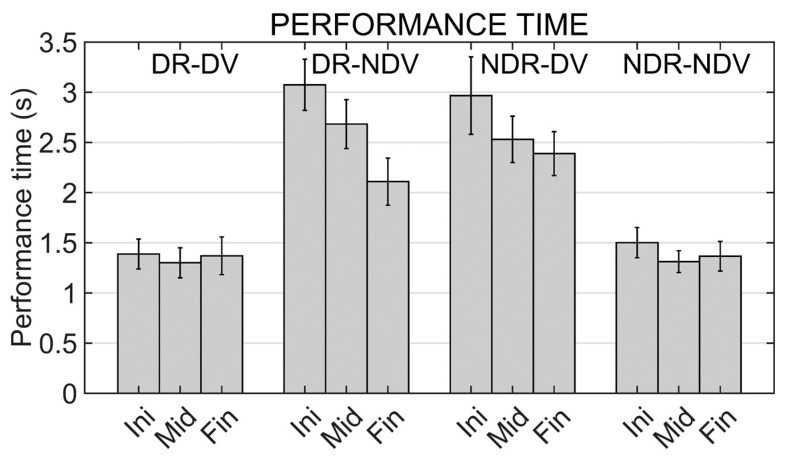
Performance times in the different experimental conditions (DR_DV, DR_NDV, NDR_DV and NDR_NDV) and in the different moments of the training (Initial, Middle and Final moments). Mean values and standard errors of the mean are shown.

Mean performance times in the different experimental conditions and moments are shown in Figure 5.

### 3.7. Trajectory Lengths

**Figure 6:**
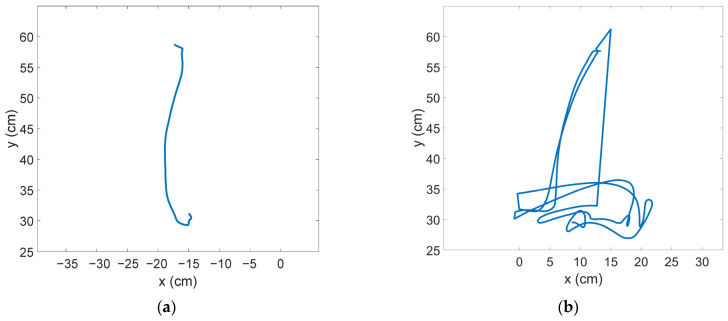

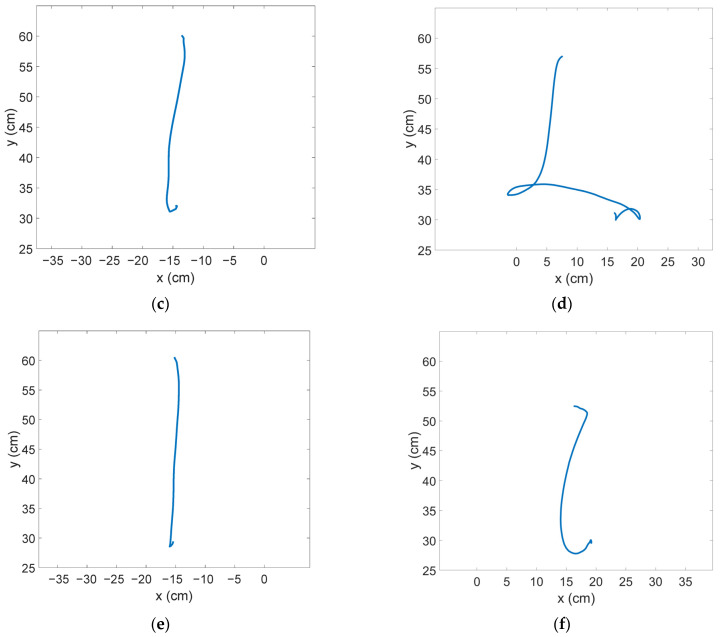
Trajectory followed by the hand in different experimental conditions and iterations in a sample subject (**a**) DR_DV, repetition 1; (**b**) DR_NDV, repetition 1; (**c**) DR_DV, repetition 6; (**d**) DR_NDV, repetition 6; (**e**) DR_DV, repetition 15; (**f**) DR_NDV, repetition 15.

Trajectories from specific repetitions (1, 6 and 15) from one of the participants are shown in Figure 6, for the DR_DV condition and for the DR_NDV condition.

Results from the two-way repeated measures ANOVA applied to trajectory lengths show a significant effect for the experimental condition (F (3,42) = 5.242; *p* < 0.004). No significant effect was found for the other two factors (moment and interaction term). Pairwise comparisons using the Bonferroni correction showed significant differences between NDR_NDV and NDR_DV (*p* < 0.017).

**Figure 7:**
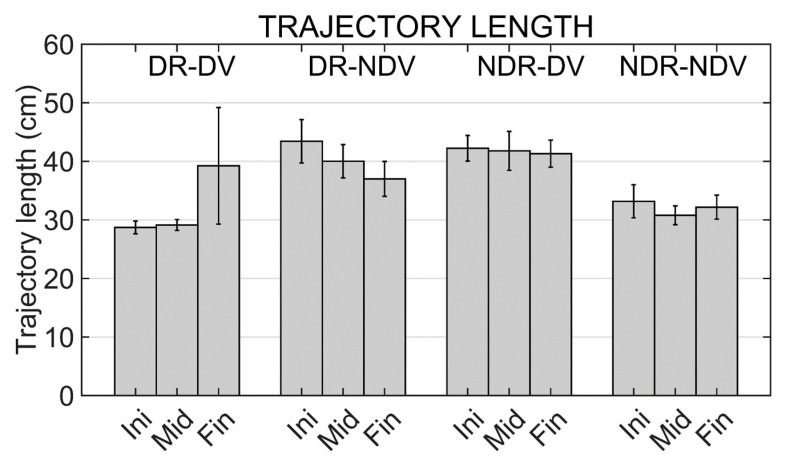
Trajectory lengths in the different experimental conditions (DR_DV, DR_NDV, NDR_DV and NDR_NDV) and in the different moments of the training (Initial, Middle and Final moments). Mean values and standard errors of the mean are shown.

Mean trajectory lengths in the different experimental conditions and moments are shown in Figure 7.

### 3.8. Maximum Vertical and Horizontal Deviations

Results from the two-way repeated measures ANOVA applied to the maximum horizontal deviation show a significant effect for the experimental condition (F (3,42) = 21.794; *p* < 0.001). No significant effect was found for the other two factors (moment and interaction term).

Pairwise comparisons using the Bonferroni correction showed significant differences in all comparisons between a mirror condition and a non-mirror condition: DR_DV and DR_DNV (*p* = 0.008), DR_DV and NDV_DV (*p* = 0.002), NDR_NDV and DR_NDV (*p* = 0.001), and NDR_NDV and NDR_DV (*p* < 0.001).

Results from the two-way repeated measures ANOVA applied to the maximum vertical deviation show no significant effect for any of the factors.

**Figure 8:**
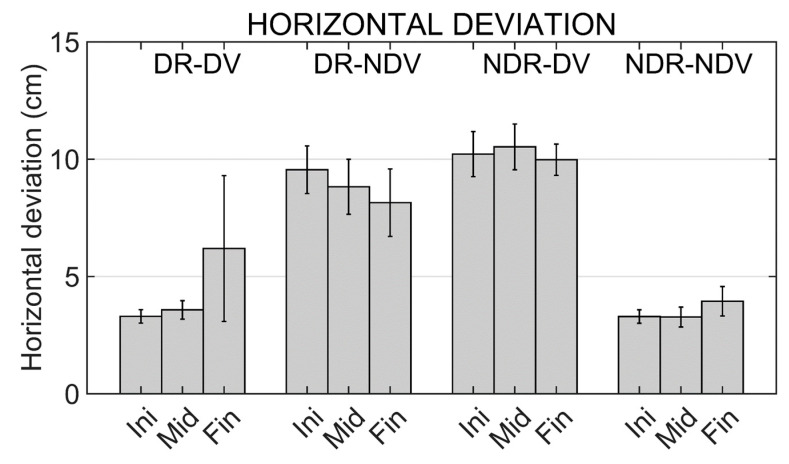
Trajectory maximum horizontal deviation in the different experimental conditions (DR_DV, DR_NDV, NDR_DV and NDR_NDV) and in the different moments of the training (Initial, Middle and Final moments). Mean values and standard errors of the mean are shown.

**Figure 9:**
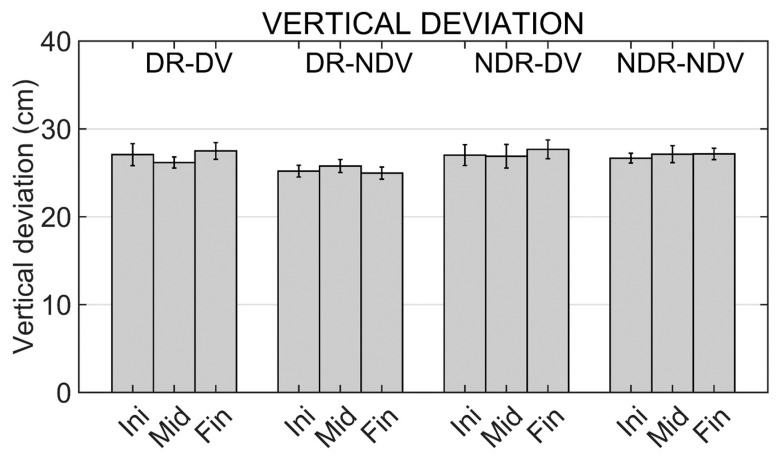
Trajectory maximum vertical deviation in the different experimental conditions (DR_DV, DR_NDV, NDR_DV and NDR_NDV) and in the different moments of the training (Initial, Middle and Final moments). Mean values and standard errors of the mean are shown.

Mean values of maximum horizontal and vertical deviations of the trajectories in the different experimental conditions and moments are shown in Figure 8 and Figure 9.

### 3.9. Presence and Usability

Results from the presence questionnaire show a median SUS-Mean value of 5.11, with an interquartile range of 1.05. The median SUS-Count value was 2 with an interquartile range of 3. Subjective evaluation of the system from the presence point of view in the final open question showed comments such as the following: “The environments are really nice, maybe the illumination of the lamp in the ceiling can be improved”; “The hands help you to feel present in the environment”; “Textures, lighting and object shadows help you to have the feeling of physically being in the room. Maybe at the end, thinking that everything was too geometrical removed a degree of realism. But most of the time I felt present in the room”; “The bookshelf gave a lot of realism to the room, as you could perceive the depth. The system would be more realistic if the complete body could be visualized”.

Results from the System Usability Scale show a mean score of 73.375 with a standard deviation of 13.40.

## 4. Discussion

In the present work, a portable VR-based MVF system has been designed and developed. The application can be run in a specific head mounted display (Meta Quest) that does not require a PC or any additional hardware to run. Consequently, the system can be easily applied in the context of home-based training.

Although technically it would be possible to add additional trackers to detect the motion of other parts of the body (such as the elbow and shoulder) to give more precision to the system and contribute to generate greater embodiment, that will also add complexity and reduce portability, making it less suitable for home-based rehabilitation or training. Consequently, the system just focuses on the tracking of hand movements and gestures, which is also the case in VR-based MVF systems that can be found in the literature [24,25,28,29].

Most of the previous VR-based MVF systems were based on head-mounted displays that needed to be connected to a computer [24,25,27,28,29,33,39] and required their use in special facilities (hospital, rehabilitation centers, universities). The current system does not need a computer, making it more appropriate for home-based training. At the moment of writing this document, the current commercial version of the required hardware (Meta Quest) has a cost of 349 EUR that can be afforded by the patient/participant or the clinic/training center depending on the specific situation and conditions.

Usually, VR-based MVF systems are based in self-developed software with simple training exercises such as wrist or fingers flexions and extensions, pinches or turns, finger movements or virtual blocks movements [24,25,28,34]. A similar approach has been followed in the present work, programming a simple motor task (moving an object from an initial to a final position) that must be performed repetitively, which is considered a critical component for neurological recovery [29,44].

The current application has been programmed to save information about the participant’s performance of the proposed exercises. On one hand, temporal information associated to the performance of each task is monitored, such as the response time (time elapsed since the presentation of a task until the participant starts to perform it) and the performance time (time required to complete the task). On the other hand, the trajectory followed by the participant’s hand to solve the task is also saved. All these data provide information that can be relevant for the trainer, especially in the context of remote training in which the trainer is not present while the participant performs the exercises. With this information, the trainer can evaluate the performance. In fact, the relevant behavioral parameters could even be integrated in an analytics platform that the trainer can use to graphically evaluate their evolution. Based on this information, recommendations can be given about the next steps to perform in the training procedure. This is one of the main contributions of the present work. Other studies have remarked upon the importance of evaluating the evolution of patients during the virtual reality training [29], but they made their analyses based only on observation. In our case, behavioral data is automatically obtained and can be analyzed off-line or on-line, if needed.

A summary of the contributions of the present study in comparison with the discussed related studies can be found in Table 3.

In the following paragraphs, the validation that has been conducted with a group of healthy participants will be discussed. Globally, results show a clear influence of the experimental condition (when comparing mirror and non-mirror conditions) on the behavioral parameters, and some indicators that the repetitive training also has an influence on the temporal responses, reducing the differences between conditions. This supports the future use of the developed virtual environment in studies with patients to validate its application for rehabilitation purposes.

Embodiment in the different experimental conditions was evaluated by means of the pESQ [37]. This questionnaire was selected because it is short and can be easily integrated in the VR experience. Besides, it allows the evaluation of the three factors of embodiment that are usually considered in the literature: self-location, agency and body ownership. The observed level of embodiment was significantly higher in any non-mirror condition than in any mirror condition. The origin of the lower level of embodiment in the mirror conditions can be explained by the self-location component. In the mirror conditions, the virtual hand is not located in the expected location, generating a lower value in the self-location component of embodiment, and consequently, a lower global evaluation of the embodiment. The body ownership and agency components are not affected by the fact of being in a mirror condition.

Continuing with the questionnaire data, mainly positive reactions to the developed environment have been reported without significant differences between experimental conditions (positive reactions in more than 80% of participants in each condition). The percentage of adverse reactions was small and a significant influence of the experimental condition on the percentage of participants that had an adverse reaction was observed. In any case, no important adverse reactions were observed and participants who reported a minor dizziness were able to finish the procedure without any disturbance.

Regarding behavioral data, low response and performance times mean values were obtained (always lower than 3.1 s). In any case, the obtained values are significantly higher in any mirror condition when compared with any non-mirror condition. This is coherent with the mirror task nature. Participants have more difficulty controlling their virtual hand in mirror conditions, and they need more time to grab the cube or to perform the movements required to translate the cube from its initial to its final location.

Mazzola et al. [28] also evaluated the time needed to perform a similar VR motor task (a block stacking task) with the dominant hand, obtaining a mean value of 2.766 s in the non-mirror condition and a mean value of 7.599 s in the mirror condition. The temporal responses that have been observed in the present study are coherent with the results of this previous one, with significantly higher values in the mirror conditions. Although Mazzola et al. [28] instructed the participants to use the dominant hand, in the present study additional experimental conditions have been considered to evaluate the performance when the non-dominant real hand is used. No differences have been observed between the task performance with the dominant and non-dominant hands. Significantly higher temporal values have been obtained in the mirror conditions both with the dominant and the non-dominant hand.

Continuing with the trajectory analyses, we can remark that the higher response and performance times observed in the mirror conditions seem to be associated to more erratic and complex trajectories. The observed trajectories in the non-mirror conditions are almost linear since the first repetition. However, in the mirror conditions more fluctuations are observed when graphically observing the trajectories. Results show a significant effect of the experimental condition on the trajectory length and on the maximum horizontal deviation.

In the case of the trajectory length, pairwise comparisons only show a significant difference between conditions when comparing the lengths observed in the mirror and non-mirror conditions if the movements in the real world are performed with the non-dominant hand. However, significantly higher values are observed in the maximum horizontal deviation when any mirror condition is compared with a non-mirror one.

There are no significant differences in the maximum vertical deviation between experimental conditions because participants find it less difficult to control movements in this dimension even in the mirror conditions. The maximum vertical deviation is approximately equal to the distance between the initial and final cube positions.

On the other hand, we expected a temporal evolution of the recorded parameters during the repetitive training in each condition. This evolution has been observed, but only in the performance time. A global reduction in the performance time has been observed between the initial part of the experiment and the middle and final parts. After performing the initial repetitions, the time to finish the task is reduced.

Besides, when the analysis between experimental conditions is performed separately in the three temporal moments of the experience, significant differences between mirror and non-mirror conditions are only present in the initial and middle parts. In the final part, significant differences are only observed between the mirror condition that is controlled by the non-dominant hand and any of the non-mirror conditions.

The results from the validation show that the system has a good usability and generates a high level of presence in the participants. The subjective comments of the participants about their presence level are coherent with the questionnaire marks. The comments show that the participants liked the environment and the textures, lighting and object shadows, although some details in specific objects such as the lamp can be improved. The hands help them to feel present in the environment, although a user indicates that it would be improved if the complete body were visualized. That is a factor that will be considered in future studies and also evaluated from the embodiment point of view. System usability was also positively evaluated, with a mean score of 73.375 in the System Usability Score. Scores beyond 68 on this scale are classified as above average [42]. Twelve of the participants scored higher than this threshold. Although lower values were observed in eight of the participants, these scores were always higher than 55, which can be considered a correct usability [42].

The present study has some limitations that are described in this paragraph. Firstly, the use of the system for rehabilitation purposes has still to be validated. The system has been developed as a generic set-up that allows the application of the MVF procedure without focusing on a specific kind of final user and has been tested with healthy participants. Secondly, the number of participants that have been included in the testing conducted in this work is reduced. Future studies to validate the use of the system for rehabilitation purposes should consider a greater group size and the inclusion of different groups of patients. These studies will evaluate how specific groups of patients use the system and, if needed, will make adaptations in the set-up according to these patients’ characteristics. The current system could be useful for different kinds of patients such as chronic pain patients or stroke patients, and that will be the next step in its validation.

VR-based MVF has advantages when compared with traditional MVF [18]. It allows the combination of the visual modality with other senses information, which can facilitate neuroplasticity in the patients. It also allows configuration of the difficulty and features of the training task, giving more versatility to the procedure. Finally, VR motivates patients and engages them in the training tasks, thus reinforcing treatment adherence.

VR allows many other configurations and that can be afforded in future works, with other tasks (including real world tasks), other training durations, other feedback modalities and different hand combinations. Virtual hands can also be personalized according to the participant’s physical characteristics [29]. VR systems also allow to modify parameters (offset, delay) in the movements of the virtual hands [45,46]. These kinds of modifications could be easily performed in the context of VR-based MVF systems and would allow interventions that would not be possible with traditional MVF. Future studies will have to analyze which configurations and training parameters are more suitable for each kind of patient and evaluate their impact on the success of the protocol.

## 5. Conclusions

The present work has designed, developed and tested a portable VR-based MVF that can be applied for home-based rehabilitation. Behavioral parameters (response time, performance time, trajectories) about the performance of the training task are monitored. Testing the system with healthy volunteers has shown that these parameters provide relevant information adequate for differentiating between different experimental conditions and evaluating the temporal evolution of the participant’s task performance. The observed results confirm the relevancy of the selected parameters and support the possibility of conducting studies with patients to validate the use of the system for rehabilitation purposes.

## Figures and Tables

**Table 1:** Median pESQ values, including global score and subcomponents of embodiment: self-location (pESQ-SL), body ownership (pESQ-BO) and agency (pESQ-A).

	DR_DV	DR_NDV	NDR_DV	NDR_NDV
pESQ	4.75	4	4	4.67
pESQ-SL	5	3	3	5
pESQ-A	4.75	4.5	4.5	5
pESQ-BO	5	4	5	5

**Table 2:** Percentage of participants with positive and adverse reactions after each experimental condition. The percentage of participants per specific reaction is also shown.

Reactions (%)	DR_DV	DR_NDV	NDR_DV	NDR_NDV
**Positive**	**95**	**80**	**80**	**80**
Fun	55	55	45	60
Relaxing	45	25	20	35
Challenging	10	25	45	5
Distracting	35	15	20	20
Others	0	0	5	5
**Adverse**	**0**	**25**	**20**	**5**
Dizziness	0	10	10	5
Fatigue	0	15	5	0
Anxiety	0	5	5	0
Others	0	0	5	5

**Table 3:** Comparison of MVF systems developed in different studies, including the system developed in the current work. The devices used in each system are specified, indicating if the developed system is portable and configurable. The training exercise is detailed. If behavioral information is monitored, it is indicated.

Study	Device	Portable	Configurable	Exercise	Behavioral Information
Current study	Meta Quest	Yes	Yes (different mirror conditions and timings)	Moving an object	Response time, performance time, trajectory
Lin et al. [24]	Oculus Rift + Leap Motion	No	No	Hand rehabilitation related activities	No
Osumi et al. [25]	Oculus Rift DK2 + Kinect + Leap Motion	No	No	Reach and touch a target object	No
Bullock et al. [27]	HTC-Vive	Yes, but with a simplified Google Cardboard version	No	Explore a virtual world where several tasks can be performed	No
Mazzola et al. [28]	HTC Vive Pro + Leap Motion	No	No	Stack four blocks	Performance time, EMG
Weber et al. [29]	Oculus Rift	No	Yes (avatar can be personalized)	Simple hand motion exercise, rock stacking and functional task	No
Ossmy and Mukamel [33]	Oculus Rift DK1 + 5DT Data Glove + PlayStation Eye Camera + Rehabit-Tec	No	No	5-digit finger sequence movement	Recorded movements from the glove can be visualized offline
Swee et al. [34]	Mobile-based VR headset + Kinect + Leap Motion	No	No	Pick and Place. Balance.	Time upon completion
Rutledge et al. [39]	Oculus Rift, motion sensor and pedaler	Yes (only with a compatible computer at home or with a smart-phone simplified version)	No	Simple exercises for arms and legs (e.g., clenching the fist)	No

## Data Availability

The data presented in this study are available on request from the corresponding author.
